# Intrinsic multipotential mesenchymal stromal cell activity in gelatinous Heberden’s nodes in osteoarthritis at clinical presentation

**DOI:** 10.1186/ar4574

**Published:** 2014-06-03

**Authors:** Thomas G Baboolal, Sally A Boxall, Sarah M Churchman, Conor T Buckley, Elena Jones, Dennis McGonagle

**Affiliations:** 1 Leeds Institute of Rheumatic and Musculoskeletal Medicine, University of Leeds, Chapel Allerton Hospital, Chapleltown Road, Leeds LS7 4SA, UK; 2 NIHR Leeds Musculoskeletal Biomedical Research Unit, Leeds Teaching Hospital NHS Trust, Chapel Allerton Hospital, Chapleltown Road, Leeds LS7 4SA, UK; 3 Trinity Centre for Bioengineering, Trinity Biomedical Sciences Institute, Trinity College Dublin, 152-160 Pearse Street, Dublin 2, Ireland

## Abstract

**Introduction:**

Gelatinous Heberden’s nodes (HNs), also termed synovial cysts, are a common form of generalized osteoarthritis (OA). We sought to determine whether HN cases at clinical presentation contained multipotential stromal cells (MSCs) and to explore whether such cells were more closely related to bone marrow (BM) or synovial fluid (SF) MSCs by transcriptional analysis.

**Methods:**

At clinical presentation, gelatinous material was extracted/extruded from the distal phalangeal joint of OA patients with HNs. From this, plastic adherent cells were culture-expanded for phenotypic and functional characterization and comparison with BM- and SF-MSCs. Mesenchymal related gene expression was studied by using a custom-designed TaqMan Low Density Array to determine transcriptional similarities between different MSC groups and skin fibroblasts.

**Results:**

In all cases, HN material produced MSC-like colonies. Adherent cultures displayed an MSC phenotype (CD29^+^, CD44^+^, CD73^+^, CD81^+^, and CD90^+^ and CD14^-^ CD19^-^, CD31^-^, CD34^-^, CD45^-^, and HLADR^-^) and exhibited osteogenic, chondrogenic lineage differentiation but weak adipogenesis. Gene cluster analysis showed that HN-MSCs were more closely related to SF- than normal or OA BM-MSCs with significantly higher expression of synovium-related gene markers such as bone morphogenic protein 4 (*BMP4*), bone morphogenetic protein receptor type 1A (*BMPR1A*), protein/leucine-rich end leucine-rich repeat protein (*PRELP*), secreted frizzled-related protein 4 (*SFRP4*), and tumor necrosis factor alpha-induced protein 6 (*TNFAIP6*) (*P* <0.05).

**Conclusions:**

Gelatinous HNs derived from hand OA at clinical presentation contain a population of MSCs that share transcriptional similarities with SF-derived MSCs. Their aberrant entrapment within the synovial cysts may impact on their normal role in joint homeostasis.

## Introduction

Osteoarthritis (OA) represents a common age-related disease characterized by progressive joint destruction, loss of function, and joint failure. Decompensated joint remodeling with florid new bone formation is also a feature of advanced OA, and generalized nodal OA represents a striking example of this bone-forming phenotype. Nodal OA may present acutely with painful joint swelling or gelatinous synovial cysts, known as Heberden’s nodes (HNs), which subsequently are associated with ossification and radiographic features of OA [1,2]. As such, HNs are seen as predictors of osteophyte formation, cartilage loss, and joint space narrowing [3,4].

Skeletal repair, remodeling, and new bone formation are thought to be linked to multipotential stromal cell (MSC) function, whereby highly proliferative cells from a variety of sources (such as bone marrow (BM), synovium, and adipose tissue) can form mesenchymal lineage tissues [5]. Consequently, substantial academic and industrial investment has focused on determining the role of MSCs in OA pathology and the potential of MSC-based therapy to treat advancing disease, and 28 clinical trials to date have used MSCs to treat OA (search terms “mesenchymal stem cells” AND “osteoarthritis” [6]).

We originally identified a population of MSCs in knee synovial fluid (SF) and observed their increase in early knee OA [7]; similarly, increases are also reported following joint injury [8,9]. It is speculated that mobilization of MSCs from the synovium into the SF is a response to tissue injury, suggesting that SF-MSCs play a role in joint homeostasis [9,10]. There is also good evidence that, under a corrected mechanical environment, using joint distraction, cartilage regeneration can be achieved without direct manipulation of the synovial compartment [11,12], again suggesting that the joint has the intrinsic capacity to restore and remodel OA-associated pathology [13]. Given that HNs are associated with new bone formation and that the constituents of the gelatinous material are similar to SF [2,14], we hypothesized whether HN cysts contain a population of MSCs similar to those found in other synovial joints.

Although ossified HNs are common, early manifestations with gelatinous material are infrequent and rarely present to the clinician at a stage when they can be aspirated [1,2]. We undertook cellular and transcriptional evaluation of culture-expanded cells from patients with gelatinous HNs at clinical presentation and compared those cells with MSCs derived from OA SF and BM from OA and normal patients. Herein, we show that such patients with early HNs have an MSC population entrapped within these cysts.

## Methods

### Collection of gelatinous material from Heberden’s nodes

**Figure 1:**
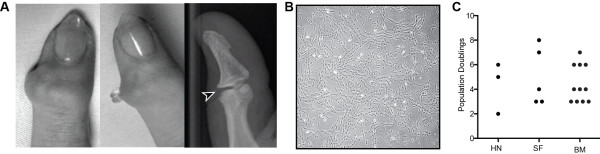
**Sample collection, cell growth, and multipotential stromal cell characterization. (A)** Distal interphalangeal joint with osteoarthritis and an acute Heberden’s node (HN) with extrusion of gelatinous material and plane radiograph demonstrating visible joint space (arrowhead) indicating preservation of articular cartilage at clinical presentation. **(B)** Example of multipotential stromal cell-like colony grown from gelatinous cyst material and **(C)** population doublings of HN cells between passages 0 and 1 compared with synovial fluid (SF)- and iliac crest bone marrow-multipotential stromal cells. BM, bone marrow.

We identified three female patients (ages 44 to 66 years) with painful distal interphalangeal (DIP) joint HNs. All imaging and samples were collected following informed written consent under ethics approved by the Leeds East NHS Research Ethics Committee. Patients had conventional x-ray of their hands prior to sample collection (Figure 1A). HNs were punctured aseptically with a 16-guage needle; gelatinous material within was extruded from the joint (Figure 1A) and collected in 5 mL of saline.

### Cell isolation and culture expansion

Extruded HN material was diluted (1:4) with non-hematopoetic (NH) expansion medium (Miltenyi Biotec, Bisley, UK), centrifuged, and resuspended in NH medium. Samples were plated in triplicate and cultured for 14 days with twice-weekly media changes in NH medium before counting colonies; these cells were considered passage 0 (p0). Thereafter, colonies were trypsinised (0.25% trypsin; Invitrogen, Paisley, UK) and further expanded. For expansion, cells were seeded at a density of 10^4^ cells per cm^2^, with twice-weekly media changes, and further grown to between p1 and p3.

For comparative analysis, MSCs were also expanded from OA knee SF aspirates as described above for HN cells (SF, n = 5 donors) and normal and OA BM. Briefly, normal iliac crest BM (ICBM) (n = 11) was aspirated from orthopedic patients undergoing elective surgery for the removal of metalwork. BM from OA patients—osteoarthritic femoral canal BM (OAFCBM) (n = 3)—was aspirated from the medullary canal at the neck of the femur during hip arthroplasty [15]. Plastic adherent cells from normal and OA BM were expanded as for NH cells. Additionally, MSCs were isolated from osteoarthritic femoral head BM (OAFHBM) (n = 3). Femoral heads from hip arthroplasty of patients with OA were mined by using a bone mill. Bone fragments were washed in phosphate-buffered saline, and cells were enzymatically extracted with collagenase as previously described [16]. For ethical reasons, BM could not be collected from the iliac crest of patients with OA. Patients were between 39 and 76 years old. All cells were seeded and expanded as above. Primary fibroblast (FB) lines CRL-2068 and HFF1 (ATCC, Teddington, UK) and NHDF (Lonza, Basel, Switzerland) were purchased and grown similarly. Growth kinetics for HN cells was compared with SF- and ICBM-MSCs by calculating the population doubling rate (PDR) between p0 and p1 by using the following equation: PDR = days in culture/population doubling (PD), where PD = log2x(log cells harvested/log cells seeded).

### Trilineage differentiation

Trilineage differentiation assays were performed as previously described [17]. Briefly, cells (2.5 × 10^5^) were pelleted and cultured in chondrogenic medium for 21 days (reagents and methods as defined in [18]). For histology, pellets were embedded in paraffin and 8-μm sections were stained with 1% toluidine blue (Sigma-Aldrich, Gillingham, UK). For osteogenic and adipogenic differentiation, cells were plated in six-well plates at a density of 10^3^ cells per cm^2^ and cultured for 21 days in either osteogenic or adipogenic differentiation media [18]; cells were stained with alizarin red and oil red O, respectively.

### Phenotypic analysis by flow cytometry

Flow cytometry was performed by using an LSRII four-laser flow cytometer (BD Biosciences, Oxford, UK) with appropriate isotype controls. Passaged MSCs (p3 from HN, SF, and ICBM) (n = 3 for each group) were stained with combinations of the following antibodies at the dilution recommended by the manufacturers: anti-CD14-allophycocyanin-cyanine (APC-H7), anti-CD19-fluorescein isothiocyanate (FITC), anti-CD34-peridinin chlorophyll protein (PerCP), anti-HLADR-phycoerythrin-cyanine (PE-Cy7), anti-CD73-phycoerythrin (PE), anti-CD90-PE, anti-CD81-FITC, anti-CD44-FITC, and anti-CD29-FITC (all from BD Biosciences). Cells were stained with 4′,6-diamidino-2-phenylindole (DAPI) (Sigma-Aldrich) as a live/dead discriminator immediately prior to acquisition [15]. At least 10,000 live cell events were collected for each antibody combination.

### Quantitative real-time polymerase chain reaction

RNA was isolated from p1- to p3-expanded MSCs from HN, SF, OAFCBM, OAFHBM, ICBM, and primary FBs (n = 3 for each group) by using the Norgen Biotec RNA/DNA/protein kit (Geneflow, Lichfield, UK) in accordance with the instructions of the manufacturer. RNA was reverse-transcribed by using the High Capacity cDNA reverse transcription kit for use on a 96-gene custom designed TaqMan Low Density Array (Applied Biosystems, Warrington, UK). Based on recommendations of the manufacturer, 200 ng cDNA was used per port (two ports per sample). Analysis used the 2^(-ΔCt)^ method, normalized to the reference gene *HPRT1*[19]. Hierarchical cluster analysis of quantitative polymerase chain reaction (qPCR) data was log2-transformed and filtered (filter = 80% present), resulting in 88 out of 96 genes analyzed. Complete linkage analysis was performed and dendrograms were generated by using Cluster 3.0 software and Java TreeView version 1.1.6 [19,20]. Statistical analysis (Mann-Whitney *U*) was performed by using SPSS 20 (IBM Inc., Portsmouth, UK).

## Results

### Gelatinous material from Heberden’s node contains multipotential stromal cell-like cells

Similar to control SF and ICBM [17], MSC-like cells were isolated and expanded from the gelatinous material from the DIP joint HNs of all three patients (Figure 1A and B). After 14 days of proliferation and from approximately 0.5 mL of gelatinous material, four, 16, and 42 colonies were present from the three patient samples. With further expansion after p0, HN cells continued to grow at rates similar to SF- and ICBM-derived MSCs (Figure 1C). Each HN culture was taken to at least p2, corresponding to approximately 15 to 20 population doublings (PDs). This was equivalent to approximately 1 PD every two days, consistent with our previously reported results for SF- and ICBM-MSCs [7].

### Trilineage potential of Heberden’s node cells

**Figure 2:**
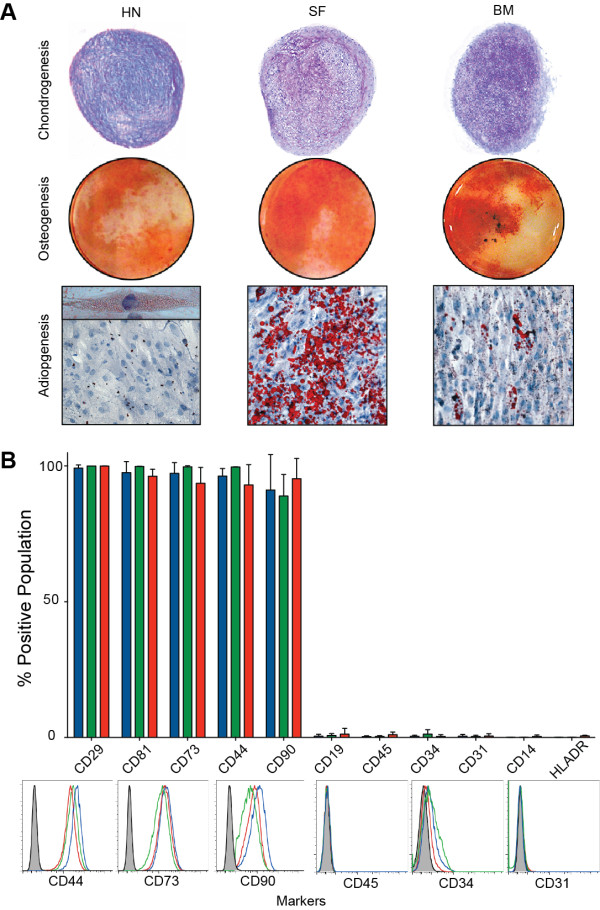
**Multipotential stromal cell (MSC) differentiation and immunophenotyping. (A)** Representative images show trilineage differentiation capacity of Heberden’s node (HN)-MSCs compared with synovial fluid (SF)- and iliac crest bone marrow (ICBM)-MSCs. Adipogenesis of HN-MSC was low, and only micro-vesicles were observed (insert). **(B)** Plot showing mean percentage of populations positive for cell surface markers by flow cytometry (n = 3 for HN-, SF- and ICBM-MSCs). Error bars represent standard deviation. Below are example histogram overlays of Heberden’s node (blue), synovial fluid (green), and bone marrow (red) MSCs with isotype control (grey-filled).

Trilineage differentiation was performed on culture-expanded (p2-3) cells (Figure 2A). Pellets formed in chondrogenic medium stained positive for sulphated-GAGs, consistent with SF and ICBM controls and indicative of progression toward the chondrogenic lineage. HN cells cultured in osteogenic medium exhibited strong alizarin red staining, similar to SF-derived MSCs but less than those derived from ICBM, indicating that cells were committed to an osteogenic lineage. In contrast to SF- and ICBM-MSCs, all HN samples tested demonstrated weak staining for adipogenesis, with only isolated cells having an accumulation of micro-vesicles (Figure 2A).

### Culture-expanded cells from Heberden’s node have a multipotential stromal cell phenotype

Flow cytometry was used to determine the immunophenotype of expanded HN cells and compared with SF- and ICBM-derived MSCs. HN cells were uniformly negative for CD14, CD19, CD31, CD34, CD45, and HLADR (Figure 2B). Consistent with an MSC phenotype, HN cells were uniformly positive for CD29, CD44, CD73, CD81, and CD90 (Figure 2B). This immunophynotype was similar to culture-expanded SF- and ICBM-MSCs.

### Quantitative real-time polymerase chain reaction shows Heberden’s node-MSC are transcriptionally related to synovial fluid-MSC

**Figure 3:**
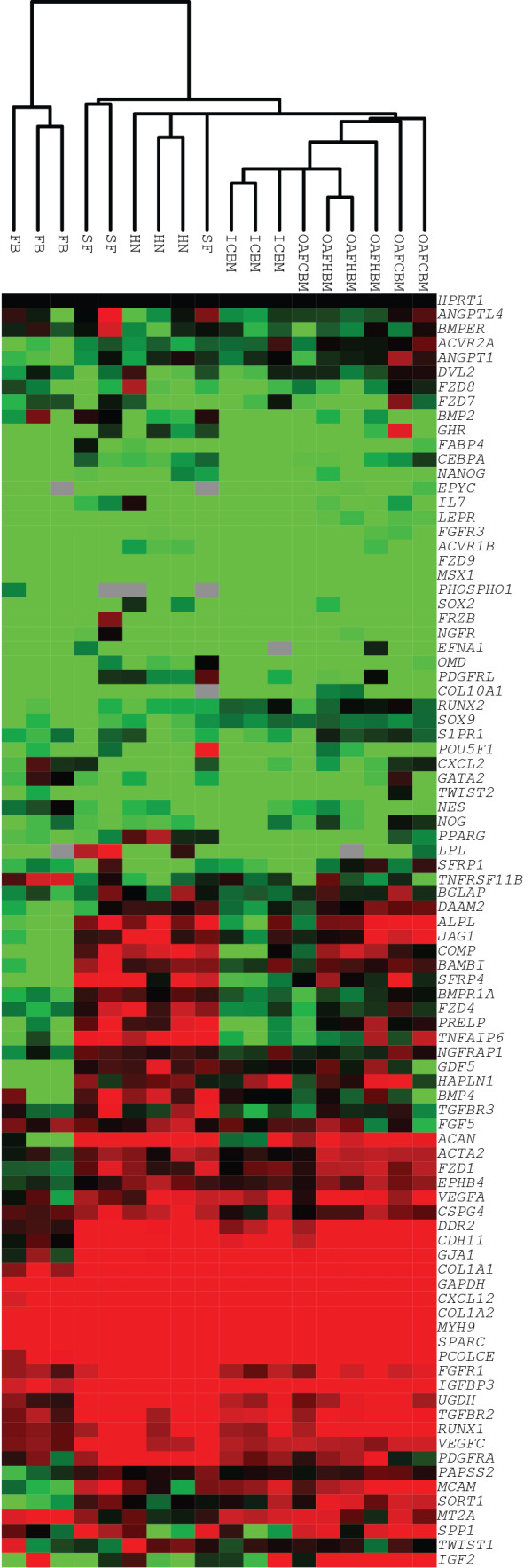
**Differential gene expression analysis.** Hierarchical cluster analysis of quantitative polymerase chain reaction data from culture-expanded Heberden’s node, synovial fluid, osteoarthritic femoral canal bone marrow, osteoarthritic femoral head bone marrow, and normal iliac crest bone marrow-derived multipotential stromal cells together with fibroblasts. Expression levels are normalized to *HPRT*1, where black is 1, red is greater than 1, green is less than 1, and grey is below detection.

To further characterize HN-MSCs, qPCR was performed by using transcripts that reflect the stromal origin and trilineage potential of MSCs [19]. Hierarchical cluster analysis was used to determine similarities in expression patterns between HN-, SF-, OAFCBM-, and OAFHBM-MSCs (all from patients with established OA) as well as MSCs from normal ICBM and primary FBs (Figure 3). MSCs derived from OA patient BM were used to control for potential differences in expression patterns between normal and OA BM. All MSC samples clustered away from FBs on a separate branch, confirming that gene expression profiles were cell type-specific (for MSCs). HN-MSCs clustered together with SF-MSCs, whereas all BM-derived MSCs clustered as a distinct group away from SF- and HN-MSCs (Figure 3). This clustering of SF- and HN-MSCs shows a similar transcriptional profile, indicating that these cells may share a common niche-specific origin and that the expressions of these genes were not related to OA.

**Figure 4:**
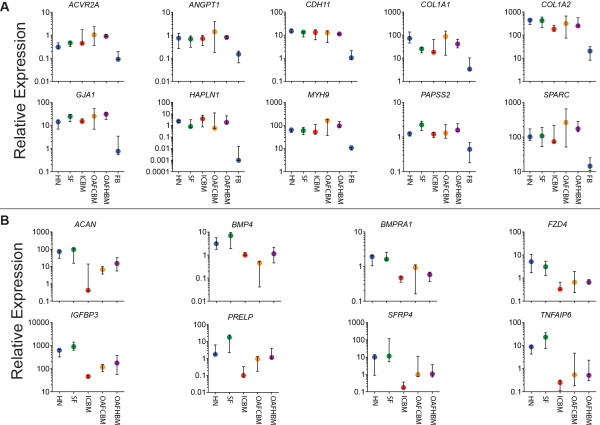
**Examples of differentially expressed genes. (A)** Relative expression levels of genes differentially expressed between all multipotential stromal cells (MSCs) and fibroblasts (FBs). **(B)** Relative expression of differentially expressed gene between Heberden’s node (HN)- and synovial fluid (SF)-MSCs compared with bone and bone marrow-derived MSCs from normal and osteoarthritis patients. All cell types were tested in triplicate. OAFCBM, osteoarthritic femoral canal bone marrow; OAFHBM, osteoarthritic femoral head bone marrow.

Examples of differentially expressed genes between MSCs and FBs are shown in Figure 4A and Table 1. These data confirmed ICBM-MSC specificity for these genes as reported in our previous study [19] and extend this to SF- and HN-MSCs, suggesting MSC specificity for these genes regardless of the tissue of origin. Additionally, we identified genes differentially expressed between MSCs from the joint and BM (Figure 4B and Table 2). Significantly, a common niche identity for both HN- and SF-MSCs was clearly seen within this set of genes as increased expression of transcripts known to be associated with synovial MSCs, such as bone morphogenic protein 4 (*BMP4*), bone morphogenic protein receptor type 1A (*BMPR1A*), protein/leucine-rich end leucine-rich repeat protein (*PRELP*), secreted frizzled related protein 4 (*SFRP4*), and tumor necrosis factor α-induced protein 6 (*TNFAIP6*) [9,21-23].

**Table 1:** Common multipotential stromal cell genes

		**Fold changing in expression between:**		
**Gene**		**HN/FB**	**SF/FB**	**ICBM/FB**
*ACVR2A*	Activin A receptor, type IB	2.8	3.5	7.0
*ANGPT1*	Angiopoietin 1	5.3	4.6	4.9
*BAMBI*	Bone morphagenietic protein and activin membrane-bound inhibitor homolog	18.2	36.8	10.2
*CDH11*	Cadherin 11	11.4	8.8	9.0
*COL1A1*	Collagen, type I, alpha 1	15.3	4.3	6.0
*COL1A2*	Collagen, type I, alpha 2	20.2	20.8	9.9
*CXCL12*	Chemokine ligand 12	3.1	5.4	1.9
*EPHB4*	Ephrin receptor B4	5.5	2.3	2.6
*FZD1*	Frizzled family receptor 1	4.7	11.5	4.6
*GDF5*^a^	Growth differentiation factor 5	73.5	4.04	25.5
*GJA1*	Gap junction protein, alpha 1	8.2	14.7	10.2
*HAPLN1*^a^	Hyaluronan and proteoglycan link protein 1	397.3	258.5	691.3
*IGFBP3*	Insulin-like growth factor-binding protein 3	59.5	107.8	5.1
*MYH9*^a^	Myosin heavy chain 9	6.0	6.0	6.8
*PAPSS2*	3′-phosphoadenosine 5′-phosphosulfate synthase 2	2.8	5.1	2.7
*RUNX1*	Runt-related transcription factor 1	3.0	59.0	1.9
*SFRP4*	Secreted frizzled-related protein 4	171.2	908.9	4.8
*SORT1*	Sortilin 1	4.4	8.9	11.1
*SPARC*	Secreted protein, acidic, cysteine-rich	7.0	7.0	7.1
*VEGFC*	Vascular endothelial growth factor C	3.1	10.2	1.6

Relative fold change in expression compared with fibroblasts (FBs) (*P* ≤0.05, Mann-Whitney *U*). ^a^Newly identified genes. HN, Heberden’s node; ICBM, normal iliac crest bone marrow; SF, synovial fluid.

**Table 2:** Tissue-specific multipotential stromal cell genes

		**Fold changing in expression between:**		
**Gene**		**HN/ICBM**	**SF/ICBM**	**HN/SF**
*ACAN*	Aggrecan	12.5	15.6	0.8
*ALPL*	Alkaline phosphatase	4.8	6.4	0.8
*BMP2*	Bone morphogenetic protein 2	3.1	20.8	0.1
*BMP4*	Bone morphogenetic protein 4	3.2	5.6	0.6
*BMPR1A*	Bone morphogenetic protein receptor, type IA	3.8	4.3	0.9
*CEBPA*	CCAAT/enhancer-binding protein	11.4	19.1	0.6
*COMP*	Cartilage oligomeric matrix protein	17.2	10.8	1.6
*DAAM2*	Disheveled associated activator of morphogenesis 2	2.9	2.2	1.4
*FZD4*	Frizzled family receptor 4	13.4	7.5	1.8
*GATA2*	GATA-binding protein 2	7.1	9.4	0.7
*IGFBP3*	Insulin-like growth factor-binding protein 3	11.8	21.3	0.6
*OMD*	Osteomodulin	7.8	32.0	0.2
*PPARG*	Peroxisome proliferator-activated receptor gamma	79.9	14.0	5.7
*PRELP*	Proline/arginine-rich end leucine-rich repeat protein	19.1	87.1	0.2
*SFRP4*	Secreted frizzled-related protein 4	35.7	189.6	0.2
*TNFAIP6*	Tumor necrosis factor, alpha-induced protein 6	33.4	97.8	0.3
*UGDH*	Uridine diphosphoglucose dehydrogenase	2.1	3.1	0.7

Relative fold change in expression identified as diagnostic for synovial fluid/Heberden’s node-multipotential stromal cells (SF/HN-MSCs) from statistical analysis (*P* ≤0.05, Mann-Whitney *U*). ICBM, normal iliac crest bone marrow.

Our data also identified increases in the expression of genes associated with chondrogenesis for both HN- and SF-MSCs over BM-derived MSCs (Figure 4B and Table 2), including aggrecan (*ACAN*): bone morphogenetic protein 4 (*BMP4*) and cartilage oligomeric matrix protein (*COMP*) [22,24]. Taken together, these data indicated a potential common origin/synovial niche specificity for HN- and SF-MSCs.

## Discussion

Acute HNs are a feature of pre-radiographic hand OA and these gelatinous synovial cysts are thought to be a forerunner of new bone formation [1-4]. The nature of the gelatinous material (being rich in hyaluronan [2,14]) and the new bone formation that occurs in OA at these locations lead us to the hypothesis that MSCs may become entrapped from the joint SF in such material in the earliest stages of OA. Herein, we show the presence of HN resident cells which met functional, phenotypic, and transcriptional profiles of MSCs that are closely related to SF-MSCs [9,19].

Although HNs are a common form of generalized nodal OA, clinical presentation at the acute phase, prior to ossification, is somewhat rare; only three cases have appeared in our clinic in the past five years [1,3]. Consequently, only a small number of such gelatinous cysts could be sampled. Despite these low sample numbers, the functional, phenotypic, and transcriptional data presented here are consistent with these cells being MSCs. There was, however, a surprising difference in the capacity of the HN-MSCs to differentiate toward the adipogenic lineage. Functional assays showed only isolated cells with the accumulation of micro-vesicles in each donor, despite the expression of the adipogenic transcription factors *CEBPA* (CCAAT/enhancer-binding protein) and *PPRAG*. A lack of adiopogenesis has been linked to ‘*in vitro*’ aging of MSCs, whereby the ability of cells to differentiate is reduced as the cells approach senescence [25,26]. Owing to the prolonged culture expansion period needed to expand HN-MSCs to sufficient numbers to complete our functional, phenotypic, and expression assays, our cells may have been approaching senescence, which has reduced their capacity to differentiate toward the adipogenic lineage.

Differential transcriptional analysis has previously been shown to reliably distinguish MSCs from FBs and subdivide MSCs from their tissue of origin by identifying commonly expressed genes when cells are grown under the same conditions [27-30]. Here, we used a similar experimental approach and confirm our previous findings [19], indicating mesenchymal lineage-specific expression of genes on our custom array (Table 1). We verified that *FZD1* (frizzled family receptor 1), *IGFBP3* (insulin-like growth factor-binding protein 3), *PAPSS2* (3′-phosphoadenosine 5′-phosphosulfate synthase 2), and *VEGFC* (vascular endothelial growth factor C) are differentially expressed between MSCs and FBs [30] and also newly identify genes differentially expressed between MSCs and FBs such as *GDF5* (growth differentiation factor-5) and *HAPLN1* (hyaluronan and proteoglycan link protein 1), which are involved in skeletal development, cartilage formation, and maintenance [24,31]. Further to defining the HN cells as MSCs, our transcriptional analysis indicates that these MSCs share a gene-specific expression profile more comparable to SF-MSCs. We compared HN- and SF-MSCs not only with normal ICBM-MSCs but also with BM from OA patients. For ethical reasons, OA BM could not be collected from the iliac crest of these patients. Rather, MSCs were derived from two sources: (a) the medullary canal of the femur and (b) trabecular bone of femoral head from OA patients all undergoing hip arthroplasty. We have previously described these MSCs as functional and phenotypic indistinct from their ICBM counterparts [15,16]. Such analysis allowed joint/BM-specific differences to be identified rather than differences relating to OA. Among genes expressed by HN- and SF-MSCs (but downregulated in BM-MSCs) were the recently identified marker of synovium MSCs, *SFPR4*[9], and several genes known to be associated with cartilage formation and maintenance, such as *ACAN*, *BMP4*, *PRELP*, and *TNFIP6* (Figure 4 and Table 2) [22,24,31,32]. To fully confirm the tissue origin of MSCs *in vivo* and to establish their migration patterns following injury or in disease, lineage tracing experiments in animal models are required [33]. Nevertheless, our transcript data as well as previous findings [27-30] indicate that broad tissue or niche-specific transcriptional profiles are maintained in culture and as such can be informative on the MSC ‘*in vivo*’ functionality in a given environment.

With both normal human and animal joints having resident SF-MSCs [17], it is possible that HN-MSCs are entrapped from SF extruded through sites of weakness in the joint capsule [34] and that, like the knee, small joints contain a resident SF-MSC population. Previous gene profile analysis indicates that SF-MSCs in turn originate from the adjacent synovium [8,9], and our data suggest a similar origin for HN-MSCs based on transcriptional analysis. Our data cannot exclude the possibility that HN-MSCs are BM-derived but transcriptionally ‘altered’ following their entrance into the joint environment; this is, however, unlikely as no damage to bone and hence the entry route for these MSCs can be identified at this early stage of OA. We therefore conclude that, similar to SF-MSCs, HN-MSCs are most likely of synovial origin. Although HNs are forerunners of new bone formation, no significant osteogenic bias was seen in any HN-MSC cultures (either by qPCR or differentiation assay); this may be explained by the fact that, in this study, HN material was collected early in disease, prior to ossification. As such, our data suggest that these MSCs are unlikely to have intrinsic osteogenic bias, perhaps with the aberrant new bone formation seen in HNs a result of subsequent alterations in joint biomechanics.

## Conclusions

We present the first description of MSCs in a small joint at a stage when cartilage damage is minimal (as seen by x-ray; Figure 1A). Future clinical studies will be needed to establish whether these MSCs are mere “bystanders” or in fact contribute to the aberrant remodeling and subsequent ossification seen during disease progression. Conversely, these MSCs may represent an intrinsic joint repair process that is abolished by cyst entrapment and altered biomechanics. The identification of MSCs within another as-yet-unexplored synovial compartment is further indication that joint-specific MSCs may be important for joint homeostasis [5,35]. Additional studies on the biology of SF-MSCs and whether these cells contribute to spontaneous joint repair are critical. The presence of these resident MSCs may also offer a distinct therapeutic advantage. Perhaps it may be possible to control disease progression and elicit effective joint repair by identifying patients earlier in disease, and correcting the underlying biochemical or biomechanical instability prior to ossification.

## Abbreviations

ACAN: aggrecan; BM: bone marrow; BMP4: bone morphogenetic protein 4; DIP: distal interphalangeal; FB: fibroblast; FITC: fluorescein isothiocyanate; HN: Heberden’s node; ICBM: iliac crest bone marrow; MSC: multipotential stromal cell; NH: non-hemopotetic; OA: osteoarthritis; OAFCBM: osteoarthritic femoral canal bone marrow; OAFHBM: osteoarthritic femoral head bone marrow; p: passage; PD: population doubling; PDR: population doubling rate; PE: phycoerythrin; PRELP: proline/arginine-rich end leucine-rich repeat protein; qPCR: quantitative polymerase chain reaction; SF: synovial fluid.

## Competing interests

The authors declare that they have no competing interests.

## Authors’ contributions

TB participated in the design of the study, drafting of the manuscript, and interpretation of the results; shared responsibility for data acquisition; and helped to perform data analysis. EJ participated in the design of the study, drafting of the manuscript, and interpretation of the results and helped to perform data analysis. DM participated in the design of the study, drafting of the manuscript, and interpretation of the results. SB, SC, and CB shared responsibility for data acquisition. All authors critically evaluated the manuscript and read and approved the final version.
